# Neighbourhood socioeconomic context, individual socioeconomic position, and overweight in young children: a multilevel study in a large German city

**DOI:** 10.1186/s40608-016-0106-4

**Published:** 2016-05-06

**Authors:** Steffen Andreas Schüle, Rüdiger von Kries, Hermann Fromme, Gabriele Bolte

**Affiliations:** Department of Social Epidemiology, Institute for Public Health and Nursing Research, University of Bremen, Bremen, Germany; Institute of Social Paediatrics and Adolescent Medicine, Ludwig Maximilian University, Munich, Germany; Bavarian Health and Food Safety Authority, Munich, Germany

**Keywords:** Multilevel study, Hierarchical regression, Neighbourhood, Contextual factors, Children, Overweight, Socioeconomic position

## Abstract

**Background:**

The context of the close neighbourhood environment in which children live has gained increasing attention in epidemiological research. This study aimed to investigate if contextual neighbourhood socioeconomic position (SEP) was independently associated with overweight in young children aged 5–7 years while simultaneously considering a wide range of individual socioeconomic determinants and known risk factors for overweight.

**Methods:**

Objectively measured body mass index (BMI) data from 3499 children (53 % boys and 47 % girls) from three surveys between 2004 and 2007 clustered in 18 school enrolment zones in the city of Munich, Germany, were analysed with hierarchical logistic regression models. An index of neighbourhood SEP was calculated with principal component analysis using aggregated data. Individual socioeconomic data, maternal BMI, and birth weight were collected with parental questionnaires. We analysed how much of the between neighbourhood variance of overweight was attributable to individual factors and how much was explained by neighbourhood SEP.

**Results:**

The prevalence of overweight, including obesity, was 14.1 %. In the final adjusted model low neighbourhood SEP was independently associated with overweight (odds ratio (OR) = 1.42, 95 % confidence interval (CI) = 1.00-2.00) compared to high neighbourhood SEP. On the individual level low parental education (OR = 1.99, 95 % CI = 1.49-2.65) or middle parental education (OR = 1.50, 95 % CI = 1.16-1.95) compared to high parental education and nationality of the child other than German (OR = 1.53, 95 % CI = 1.17-1.99) compared to German nationality were independently associated with overweight.

**Conclusions:**

Whereas individual determinants were the main drivers in explaining between neighbourhood variance, neighbourhood SEP additionally explained differences in overweight between neighbourhoods. Thus, considering neighbourhood context in intervention planning could result in more effective strategies compared to measures only focusing on individual determinants of overweight.

## Background

The increase of overweight and obesity in young children in middle and high income countries in recent decades is described as one of the most challenging public health problems [1, 2]. Children being overweight or obese are at greater risk of pulmonary, orthopaedic, neurological, gastroenterological, endocrine, or cardiovascular diseases in their later life [3, 4]. Therefore, a deeper understanding of the causes of overweight in younger ages and the identification of population groups which are mostly affected and need appropriate interventions is of great importance.

Socio-ecological approaches targeting supportive environments are attracting increasing attention for overweight prevention [5]. Therefore, the research question how contextual neighbourhood factors influence overweight in children is of great interest. A contextual effect is present if factors on the neighbourhood level are independently associated with individual health outcomes while possible individual level risk factors are simultaneously considered to avoid an ecological fallacy. To separate out potential contextual effects from individual effects a multilevel modelling approach offers an appropriate analytic strategy [6–8].

Recent reviews provide evidence that a low contextual neighbourhood socioeconomic position (SEP) is a good predictor for negative health outcomes in childhood. There is still a great heterogeneity in neighbourhood studies on how indicators of neighbourhood SEP are operationalized and a comprehensive theory is still missing. Measures of income, education and employment on the level of administrative areas, such as census tracts, are the most often used indicators describing neighbourhood SEP [9–12].

We used the term SEP in this paper as suggested by Krieger et al. [13, 14]. It is defined as a term which combines economic and social resources with prestige-based characteristics which relatively position individuals, households or neighbourhoods in society.

Most studies analysing associations between neighbourhood SEP and child health were conducted in the USA. Moreover, only a minority of studies have analysed how neighbourhood SEP was associated with measures of overweight in younger children while simultaneously taking into account relevant socioeconomic and parental factors on the individual level in order to disentangle their independent associations. There is evidence that parental education and occupation, household income, and household conditions are the most important socioeconomic indicators during childhood [15]. Parental overweight [16] or high birth weight [17] should be additionally considered as important adjustment variables because these factors can confound associations between neighbourhood SEP, individual SEP and overweight in young children.

Therefore, this study aimed to analyse how the socioeconomic context of neighbourhoods was associated with overweight in young children while simultaneously considering indicators of individual SEP in multilevel analysis as well as birth weight and maternal body mass index (BMI) as adjustment variables. A further objective of this study was to determine how much variance of overweight between neighbourhoods was explained by individual factors and how much was attributable to the neighbourhood socioeconomic context.

## Methods

### Study population and study area

Data collection was performed within the health monitoring units in Bavaria (GME, Gesundheits-Monitoring-Einheiten) which are organized by the Bavarian Health and Food Safety Authority. Main goal of the GMEs is to monitor health status of children in Bavaria. Therefore, in three consecutive years surveys with identical procedures concerning data collection were conducted within the framework of the obligatory school entrance health examination in three rural and three urban study regions. All parents gave their written informed consent. The ethics committee of the Bavarian medical council approved the procedures of data collection before the first survey [18]. There were only slight modifications of the questionnaires between the surveys.

This analysis considered 3499 children aged 5–7 years in one of the GME study regions, the city of Munich. Data were pooled from the three surveys conducted between 2004 and 2007 in Munich. The children were clustered in 18 school enrolment zones with a range of 117 – 331 children per school district. These districts were used as a proxy for the children’s close neighbourhood environment.

### Measures of overweight

Weight and height were objectively measured by trained staff of the local health authority. Age–specific BMI percentile curves specific for boys and girls, respectively, were used to derive cut-offs for defining children as overweight or obese. We used the International Obesity Task Force (IOTF) cut-off values by Cole et al. [19]. In our analysis the definition of overweight did also include children with obesity.

### Individual characteristics from parental questionnaires

We defined three categories of parental education. The highest level of completed education achieved either by the mother or the father was considered. ‘High’ included a final degree at university or technical college, A-levels, or advanced technical college entrance qualification. ‘Middle’ included upper secondary school certificate or adequate graduation. ‘Low’ included a lower secondary school certificate or no graduation.

Household equivalent income was calculated based on the reported monthly household net income as disposable income after taxes and social transfers weighted for age and number of household members according to the Organization for Economic Co-operation and Development-modified scale [20]. A relative poverty threshold was defined as 60 % of the median household equivalent income in Bavaria [21]. Three income groups were created: ‘low’ (<60 % of median), ‘middle’ (60 % of median – median), and ‘high’ (>median). Due to a high number of missing information on household income in our dataset we created an additional income group ‘not indicated’ including parents who did not respond on their income in order to avoid selection bias [22].

Parental working status was considered as a binary variable. Unemployment within household was applied if both parents were marginally employed at most. The category employment was applied if one parent was at least part-time employed. A binary variable of single parenthood was created by combining three answers about single parent, family status, and living together with a partner. Only responses showing consistency in all three answers were taken into account [22].

Household crowding was present if there was more than one person per room or less than 20 m^2^ per person available. Nationality of the child was used as an indicator of migration status. Following the rationale of Schenk et al. [23], categories of German nationality and non-German nationality, including dual citizenship, were defined.

Birth weight and BMI of the mother were obtained from parental questionnaires, too. Three categories of birth weight were generated using international cut-offs from the Pediatric Nutrition Surveillance System (PedNSS) by the Centres for Disease Control and Prevention (CDC) [24]: Low (<2500 gram), normal (2500 gram - 4000 gram), and high (>4000 gram). Maternal BMI data were categorized into normal (<25 kg/m^2^), overweight (25 kg/m^2^ - <30 kg/m^2^), and obese (≥30 kg/m^2^).

### Neighbourhood socioeconomic variables

We considered five aggregated variables on the level of administrative primary school enrolment zones in which the children live. Averages from the years 2006 and 2007 were calculated. From the city council of Munich we obtained data on foreigners and migration background (percentage of residents with no German citizenship, and percentage of residents with a German citizenship and a migration background), and household data (percentage of single parent households). Data on education were provided by microm GmbH, Neuss, Germany (percentage of households with lower education and with vocational training).

### Statistical analysis

All statistical analyses were performed using SAS statistical software package version 9.3 (SAS Institute, Cary, NC, USA).

We performed bivariate logistic regression between socioeconomic neighbourhood variables, individual variables and overweight. All individual variables which were associated with overweight with a Wald’s *P* <0.2 in bivariate logistic regression were included in multivariate analysis. All socioeconomic neighbourhood variables which were associated with overweight with a Wald’s *P* <0.2 were taken into account for principal component analysis (PCA). This cut-off is recommended for initial covariable selection [25].

PCA was used as a statistical procedure for data reduction of correlated variables because it creates non-correlated orthogonal linear combinations explaining the maximum of variance [26]. The first component explains most of the variance and was therefore used as an indicator for the socioeconomic neighbourhood environment. Higher values of the index imply a lower neighbourhood SEP. Spearman rank correlation coefficients between socioeconomic neighbourhood variables used for PCA and the first component were calculated to check how each neighbourhood socioeconomic indicator was represented in the index. Finally, the index was categorized into tertiles (high, middle, and low neighbourhood SEP).

The variance inflation factor (VIF) (*VIF*_*i*_ = 1/*T*_*i*_) was used to assess multicollinearity between the covariables. The VIF is calculated with the tolerance (T) (*T*_*i*_ = 1 − *R*_*i*_^2^). *R*_*i*_^2^ is the calculated variance of each covariate associated with all other independent variables. A VIF higher than 10 indicates a serious problem of multicollinearity [27–29].

We applied multilevel logistic regression modelling with school districts as random intercepts to correct for clustering of individuals within the same school district [30]. Our calculated index of neighbourhood SEP was modelled as a 2nd level variable. All individual level variables were considered on the 1st level. Multilevel modelling enables to estimate variance between school districts separately from residual variation between individuals. Thus, this modelling approach makes quantification of overweight variance between neighbourhoods being explained by our calculated neighbourhood SEP index possible. The GLIMMIX procedure in SAS was used for calculating multilevel models.

In a first empty null model only school districts were modelled as random intercepts in order to assess the covariance parameters for the random intercept variance of overweight between school districts. In a second model individual level variables were included to analyse how these variables were associated with overweight, and how much of the variance between school zones was explained by these factors. In the full third model the index of neighbourhood SEP was added to assess if there was an independent association between neighbourhood SEP and overweight. For multivariate analysis observations with missing values in any independent variable were not taken into account, except for household income. The category ‘not indicated’ was generated because of a high number of missing values for this variable. For all other variables considered for multivariate analysis the amount of missing values was acceptable (≤7 %).

Multilevel models were adjusted for the three survey years considering each survey as a dummy variable and maternal BMI and birthweight. For the neighbourhood intercept variance estimates covariance tests were performed and *p*-values and confidence intervals were calculated. Based on the neighbourhood intercept variance estimates we calculated the proportional change in variance (PCV) in percent according to the following equation by Merlo et al. [31, 32]: *PCV* = ((*V*_a_-*V*_b_)/*V*_a_) × 100. *V*_a_ is the between neighbourhood variance of the empty model and *V*_b_ is the between neighbourhood variance including covariables, in the individual model and the full model respectively. As a sensitivity analysis, we performed multiple imputation for missing values for household income. Multiple imputation of hierarchical data is still a research area with remaining issues and there is still no standard procedure to pool covariance estimates from the random intercepts [33]. Therefore, we performed multiple imputation for fixed effects only in order to check if estimates differed to our models considering missing values as an additional income category. We applied cumulative logistic regression imputation within the PROC MIANALYZE procedure in SAS which is appropriate for ordinal variables [34–36]. In order to consider our clustered data structure, school zones were taken into account as dummy variables within the imputation procedure.

## Results

### Characteristics of study population

There were 53 % boys and 47 % girls in the study population. The overall prevalence of overweight, including obesity, was 14.1 %. Sex-specific prevalence was similar in boys (14.0 %) and girls (14.2 %). 8.5 % of the children had a high birth weight and 24.2 % of mothers were overweight or obese. 17.4 % of the parents had a low education. 13.1 % of the families were affected by relative poverty with a household income below 60 % of the median Bavarian equivalent household income. In 6.9 % of households parents were unemployed, 14.4 % reported to be single parents, and 35.5 % of the families were affected by household crowding (Table 1).

**Table 1:** Characteristics of study population

	N	Percent
Individual Variables		
Overweight (N = 3499)		
Yes	494	14.1
No	3005	85.9
Sex (N = 3499)		
Boys	1856	53.0
Girls	1643	47.0
Nationality of the child (N = 3479)		
Other than German	658	18.9
German	2821	81.1
Birth weight (N = 3499)		
Low (<2.500 gram)	407	11.6
Normal (2.500 gram – 4.000 gram)	2794	79.9
High (>4.000 gram)	298	8.5
BMI mother (N = 3250)		
Normal (<25 kg/m^2^)	2464	75.8
Overweight (25 kg/m^2^ - <30 kg/m^2^)	591	18.2
Obese (≥30 kg/m^2^)	195	6.0
Parental education (N = 3380)		
High	1971	58.3
Middle	822	24.3
Low	587	17.4
Parental working status (N = 3405)		
Unemployment within household	236	6.9
At least one parent employed	3169	93.1
Single parenthood (N = 3435)		
Single parent	493	14.4
Other	2942	85.7
Eqivalent household income (N = 3499)		
Low (<60 % Median) ^a^	457	13.1
Middle (60 % to Median)	812	23.2
High (>Median)	824	23.6
Not indicated	1406	40.2
Household crowding (N = 3406)		
Yes	1210	35.5
No	2196	64.5
Contextual Variable		
Neighbourhood SEP (N = 3499)		
High	974	27.8
Middle	1150	32.9
Low	1375	39.3

*N* total number of observations, *SEP* Socioeconomic position

^a^Median equivalent household income in Bavaria

### Principal component analysis and neighbourhood SEP index

On the neighbourhood level, except of the percentage of single parent households, all other four aggregated socioeconomic variables were associated with overweight in bivariate logistic regression (Wald’s P <0.2) and were therefore used for PCA (results not shown). The four neighbourhood variables percentage of foreigners, percentage of German residents with migration background, percentage of households with lower education, and percentage of households with vocational training were significantly correlated with the neighbourhood SEP index derived from the first principal component. Spearman rank correlation coefficients ranged between 0.69 and 0.97 and had *p*-values <0.05 (results not shown). According to our calculated neighbourhood SEP index, 39.3 % of the study population lived in school districts with a low neighbourhood SEP (Table 1).

### Multilevel logistic regression

In bivariate logistic regression all variables on the individual level, except sex and single parenthood, were associated with overweight (Wald’s P <0.2). Therefore, sex and single parenthood were not included in multivariate analysis. Low parental education, parental unemployment, low household income, household crowding, and a low neighbourhood SEP were associated with children’s overweight. Maternal overweight and a high birth weight were associated with overweight, too (Table 2).

**Table 2:** Bivariate associations of individual factors and neighbourhood SEP, respectively, with overweight

Variables	OR (95 % CI)	*p*-value
Sex		
Boy	0.98 (0.81-1.19)	0.8429
Girl	Reference	
Nationality of the child		
Other than German	2.05 (1.65-2.54)	<.0001
German	Reference	
Birth weight		
Low (<2.500 gram)	0.92 (0.67-1.25)	0.5808
Normal (2.500 gram – 4.000 gram)	Reference	
High (>4.000 gram)	1.78 (1.33-2.40)	0.0001
BMI mother		
Normal (<25 kg/m^2^)	Reference	
Overweight (25 kg/m^2^ - <30 kg/m^2^)	2.44 (1.93-3.08)	<.0001
Obese (≥30 kg/m^2^)	3.08 (2.19-4.34)	<.0001
Parental Education		
Low	2.53 (1.98-3.23)	<.0001
Middle	1.76 (1.39-2.23)	<.0001
High	Reference	
Parental working status		
Unemployment within household	1.63 (1.17-2.27)	0.0042
At least one parent employed	Reference	
Equivalent household income		
Low (<60 % Median)^a^	2.35 (1.69-3.27)	<.0001
Middle (60 % to Median)	1.79 (1.32-2.43)	0.0002
High (>Median)	Reference	
Not indicated	1.68 (1.27-2.22)	0.0003
Single parenthood		
Single parent	1.02 (0.77-1.34)	0.9079
Other	Reference	
Household crowding		
Yes	1.55 (1.27-1.88)	<.0001
No	Reference	
Neighbourhood SEP		
High	Reference	
Middle	1.35 (1.03-1.77)	0.0319
Low	2.00 (1.55-2.56)	<.0001

*OR* Odds ratio, *CI* Confidence interval, *SEP* Socioeconomic position

^a^Median equivalent household income in Bavaria

Multicollinearity analysis was performed with the neighbourhood SEP index and all eligible individual variables for multivariate analysis. The values of the VIFs showed acceptable values ranging from 1.0 to 1.8 (results not shown).

In the multilevel null model there was a significant random intercept variance of overweight between neighbourhoods (*p*-value = 0.035) (Table 3). In both multilevel models containing individual level variables only (individual model, Table 3) and neighbourhood SEP additionally (full model, Table 3) low or middle parental education and non-German nationality of the child were positively associated with children’s overweight. All other characteristics describing individual socioeconomic position remained not significant. In the full model with neighbourhood SEP as a second level variable a low neighbourhood SEP was positively associated with overweight independent from individual factors.

**Table 3:** Multivariate associations between individual SEP, neighbourhood SEP and overweight applying multilevel logistic regression (*N* = 3125)

Covariables	Null model	Individual model^a^ OR (95 % CI)	Full model^a^ OR (95 % CI)
Nationality of the child			
Other than German		1.53 (1.18-1.99)	1.53 (1.17-1.99)
German		Reference	Reference
Parental Education			
Low		2.04 (1.54-2.72)	1.99 (1.49-2.65)
Middle		1.53 (1.18-1.99)	1.50 (1.16-1.95)
High		Reference	Reference
Parental working status			
Unemployment within household		1.20 (0.82-1.77)	1.19 (0.80-1.75)
At least one parent employed		Reference	Reference
Equivalent household income			
Low (<60 % Median)^b^		1.22 (0.82-1.83)	1.18 (0.79-1.77)
Middle (60 % to Median)		1.29 (0.93-1.80)	1.26 (0.90-1.75)
High (>Median)		Reference	Reference
Not indicated		1.14 (0.83-1.58)	1.12 (0.81-1.55)
Household crowding			
Yes		0.94 (0.74-1.20)	0.93 (0.72-1.19)
No		Reference	Reference
Neighbourhood SEP			
High			Reference
Middle			1.01 (0.71-1.46)
Low			1.42 (1.00-2.00)
Measures of variation			
Neighbourhood intercept variance (95 % CI)^c^	0.11 (0.046-0.48)	0.047 (0.015-0.80)	0.026 (0.005-20.37)
Proportional change in variance		−57.3 %	−19.1 %

*OR* Odds ratio, *CI* Confidence intervall, *SEP* Socioeconomic position

^a^Adjusted for survey year, birth weight, and BMI of mother, ^b^Median equivalent household income in Bavaria, ^c^Covariance parameter estimates from random intercepts on the log odds scale

The full model including neighbourhood SEP explained additional 19.1 % between neighbourhood variance of overweight. However, the neighbourhood intercept variance estimates from which the PCV was calculated showed wide confidence intervals.

Our sensitivity analysis with multiple imputed data for missing values on household income revealed similar estimates for individual variables and contextual neighbourhood SEP. Therefore, we reported our multilevel results without multiple imputation of the ordinal income variable because we would like to guarantee valid covariance parameter estimates of our variance components (see methods for further details). Moreover, we analysed potential interactions between our significant fixed estimates of our final model and no significant interactions were detected (results not shown).

## Discussion

In our final multilevel model low neighbourhood SEP was independently associated with overweight in young children. However, determinants on the individual level explained most between neighbourhood variance of overweight.

Apart from individual SEP we additionally considered birth weight and maternal BMI which are important risk factors for overweight in young children, too [16, 17]. The association between low neighbourhood SEP and overweight remained significant which strengthened the evidence of an independent impact of neighbourhood SEP on overweight in young children. To the best of our knowledge this is one of the first studies addressing this research question and additionally considering these two important risk factors in multivariate analysis.

In comparison to our findings previous multilevel studies which analysed the influence of neighbourhood SEP on overweight in younger children found an independent association between neighbourhood socioeconomic factors and overweight, too. A longitudinal study in Canadian children aged 2–11 years found out that a poor neighbourhood context based on household income was associated with increasing BMI independent from individual age, sex, education, income, and family structure [37]. Cross-sectional data from the same study which analysed children and youth from 5 to 17 years detected also higher odds for being overweight in neighbourhoods with a low SEP index calculated with data on unemployment, family income, and education [38].

One study from the USA, which analysed children aged 6–18 years, found a positive association between decreasing neighbourhood median household income and obesity and a negative association between increasing home ownership on the neighbourhood level and obesity independent from individual age, sex and SEP. As a proxy for individual SEP the insurance status was considered [39].

A study from Germany which analysed data from the school entrance examination found a positive association between a high percentage of low educational households in the neighbourhood and overweight in 6-year old children [40]. In comparison to our study, on the individual level only the mother tongue was considered as an indicator for individual SEP.

All multilevel studies we identified did not consider birth weight and parental overweight as potential adjustment variables. Moreover, there were great differences concerning the included socioeconomic factors on the individual level and the age groups being considered. Besides that, most studies considered single socioeconomic neighbourhood factors, such as measures of income, unemployment, or education, and did not combine them into an index.

Multilevel studies investigating the independent influence of neighbourhood SEP on overweight in adolescents found similar results [41–43]. A detailed discussion of these studies would go beyond the scope of this study because our study focused on younger children. Most of these studies we identified were cross-sectional and strengthened the need for longitudinal studies investigating contextual effects of neighbourhood characteristics along the life course from early childhood up to adolescence in order to disentangle individual, family, and neighbourhood relationships.

Our final multilevel model showed that 19.1 % of overweight prevalence between neighbourhoods was explained by neighbourhood SEP and most of the variance was attributed to individual factors (57.3 %). However, these estimates should be interpreted with caution because our neighbourhood intercept variance estimates of our individual and full multilevel model showed wide confidence intervals. In only two of our identified studies variance measures were reported. In the study by Grow et al. socioeconomic neighbourhood context explained around 24 % of overweight variance between neighbourhoods [39], whereas in the study by Lange et al. 40 % of BMI variation between neighbourhoods was explained by neighbourhood unemployment [41]. A systematic review by Sellström & Bremberg [12] identified multilevel studies which studied the impact of neighbourhood factors on child and adolescent health. The review calculated that across studies on average 10 % between neighbourhood variance of the health outcome was explained by contextual factors. Health outcomes in this review were mainly problem behaviours, child maltreatment, injuries, and birth weight. The number and heterogeneity of considered factors on the individual level and the diversity of socioeconomic neighbourhood indicators on the contextual level could explain the large differences of the calculated variance measures.

There are various conceptual models framing the multidimensional pathways how neighbourhood context influence individual health [44–50]. One hypothesis of all these models is that physical environmental factors mediate the effects of neighbourhood SEP on individual health. In the context of overweight, access and quality of food environments, public resources such as parks or playgrounds, and walkability of the built environment could be such potential mediating neighbourhood factors. One hypothesis derived from the environmental justice framework states that built environmental exposures are social unequally distributed both on the individual and the neighbourhood level (exposure variation by SEP) [51]. There is much evidence that a low SEP is inversely associated with a higher environmental burden [52, 53]. Thus, more studies are needed which investigate underlying mechanisms on the pathway between neighbourhood socioeconomic deprivation and overweight in early childhood.

There are some limitations within our study. One is that our study is cross-sectional. However, for the socioeconomic factors analysed in our study reverse causation is very unlikely. Furthermore, we used administrative school enrolment zones as a proxy for the neighbourhood environment. We were not able to draw inferences to what extent these administrative zones correlate with the perceived and used neighbourhood environment of the children and their parents. Besides that, there were no data available on average household income on the neighbourhood level which is a further socioeconomic indicator often considered in neighbourhood studies. Moreover, we were not able to consider other individual risk factors, such as smoking during pregnancy, breastfeeding, or data on nutrition and physical activity. However, there is evidence at least for Germany that parental overweight, high birth weight and socioeconomic indicators are the main determinants for overweight in early childhood [16], and we were able to consider all these individual determinants in our multilevel model. Finally, for analysing random-slopes and cross-level interactions 18 level 2 units might be too low. Although simulation studies showed that 18 level 2 units may be enough for hierarchical logistic regression modelling [54] our random intercept estimates should be interpreted with caution because they showed wide confidence intervals.

One of the major strengths of our study is that we could provide new evidence for the population group of younger children because there is still a lack of knowledge how contextual neighbourhood factors influence health in early childhood, especially in Germany. To the best of our knowledge it is one of the first studies for this age group in Germany analysing neighbourhood SEP simultaneously with a wide range of individual socioeconomic indicators and the additional consideration of maternal BMI and birth weight as further important individual risk factors. Our BMI measures for children were derived from objectively measured body weight and height by trained staff, thus no bias occurred because of self-reported measures by the parents.

## Conclusions

Our study showed that the socioeconomic context in which young children live was associated with overweight independently from individual overweight determinants. Although individual determinants play a more important role in explaining differences in overweight between neighbourhoods, contextual neighbourhood factors should be additionally taken into account for the identification of vulnerable neighbourhoods and population groups. Public health interventions which consider neighbourhood context could be more effective than interventions targeting only at individual risk factors.

### Availability of data and materials

The dataset supporting the results of this article may be requested from the Bavarian Health and Food Safety Authority, steering committee of the health monitoring units in Bavaria, Munich, Germany.

## Abbreviations

SEP: socioeconomic position
BMI: body mass index
OR: odds ratio
CI: confidence interval
GME: Gesundheits-Monitoring-Einheiten
IOTF: International Obesity Task Force
PedNSS: Pediatric Nutrition Surveillance System
CDC: Centres for Disease Control and Prevention
PCA: principal component analysis
VIF: variance inflation factor
PCV: proportional change in variance
